# Microstructural and histochemical characteristics of *Lycium barbarum* L. fruits used in folk herbal medicine and as functional food

**DOI:** 10.1007/s00709-018-1277-2

**Published:** 2018-06-15

**Authors:** Agata Konarska

**Affiliations:** Department of Botany, Faculty of Horticulture and Landscape Architecture, University of Life Sciences in Lublin, Akademicka 15, 20-950 Lublin, Poland

**Keywords:** Alkaloids, Chromoplasts, Essential oils, Fluorescence and ultrastructure, Goji berries, Phenols

## Abstract

*Lycium barbarum* L. fruits, referred to as functional food, have long been used in traditional and folk herbal medicine due to their therapeutic properties. The fruit microstructure was analysed using light, scanning and transmission electron microscopes. The distribution of bioactive compounds in drupe tissues was assessed with histochemical and fluorescence assays. The analysis of the microstructure has shown that the fruit is covered by a skin with an amorphous cuticle and a layer of amorphous epicuticular waxes on the surface. The skin is composed of a single-layered epidermis with thickened walls and one layer of hypodermis with slightly thickened periclinal walls. The pericarp cells contain different types of chromoplasts, which most often contained exhibited reticulotubules/fibrils of carotenoid pigments and phytoferritine deposits. The results of the histochemical assays demonstrated that the secondary metabolites with high phytotherapeutic importance were located in all layers of the pericarp and seeds and, specifically, in the drupe exocarp and endocarp. The phytochemicals were represented by polysaccharides (LBP), lipid compounds (carotenoids, essential oils, sesquiterpenes, steroids), polyphenols (tannins and flavonoids), and alkaloids. This study, which is the first report of the microstructure and localisation of bioactive compounds in wolfberries, is a valuable complement of phytochemical analyses and can be helpful for enhancement of the therapeutic effect of the fruit as well as preliminary assessment of the medicinal potential in the search for new pharmaceuticals. Detailed anatomical studies are crucial for exploration of determinants of fruit quality and useful for identification of diagnostic taxonomic traits.

## Introduction

The increase in public awareness and the frequent ineffectiveness of conventional medicine have contributed to rapid development of phytotherapy. Plants containing biologically active compounds with health-enhancing properties known for hundreds or thousands of years are experiencing a renaissance and have aroused interest in the world of science. *Lycium barbarum* L. (*L. halimicifolium* Mill.) is a small or large shrub from the family Solanaceae, subfamily Solanoideae, tribe Lycieae growing naturally in northwest and central China. This species is widespread in the Mediterranean area as well as South-West and Central Asian regions; it also occurs in the south-east of Europe (Bensky and Gamble 1993; Zhu 1998). Seventeen *Lycium* species have been reported from southern Africa, and 50–60 species can be found in the western hemisphere (Joubert 1981).

In Poland, *L. barbarum* is a domesticated and often wild-living shrub with a low decorative value (Seneta and Dolatowski 2004). Since it grows successfully on dry, infertile soils and is very resistant to frost and air pollution, it is often used for strengthening steep and dry slopes, in schemes of wasteland management, and for uncut hedges (Szweykowska and Szweykowski 2003). Additionally, *L. barbarum* has a great beekeeping value as a polleniferous and nectariferous species; it is characterised by a long flowering period (from May to September) and is willingly visited by pollinating entomofauna (Bing et al. 2010; Wang et al. 2011). *L. barbarum* are functionally dioecious plants producing male-sterile (i.e. female) and hermaphroditic plants. Fruits are produced exclusively by female specimens, whereas hermaphroditic plants function essentially as male plants (Miller and Venable 2002, 2003).

The fruit in the genus *Lycium* is a berry or drupe with a sclerenchymatous endocarp (Bernardello 1983, 1986a; Olmstead et al. 1999, 2008; Miller 2002). *L. barbarum* drupes are known as ‘Goji berries’, which is a common name for the fruits of two species, i.e. *L. barbarum* and *L. chinense.* These taxa are very closely related and the tradition of the use of their fruits in Asian countries (China, Japan, Korea, Vietnam, Thailand, and Tibet) dates back several thousand years (Jin et al. 2013). *L. barbarum* drupes are sweet, two-seeded, and quite large, whereas the smaller and slightly bitter fruits of *L. chinense* contain several seeds (Górnicka 2015). ‘Goji berries’ are regarded as functional food and are often eaten raw (fresh or dried), added to soups, processed into juices, wines, tinctures, or teas, and used as supplements in the form of powder or pills (Potterat 2010; Kulczyński and Gramza-Michałowska 2014). For a long time, consumption of *L. barbarum* fruit was assumed to cause poisoning due to the content of the atropine alkaloid (Szafer et al. 1953; Harsh 1989; Rutkowski 2006). Investigations conducted by Adams et al. (2006) and Wang (2006) have shown variable levels of atropine in wolfberry fruits but the investigators have questioned its toxic concentration.

*L. barbarum* is considered a medicinal plant used in traditional and folk herbal medicine (Bensky and Gamble 1993; Chang and But 2001; Wang 2006). Its fruit (*fructus Lycii*) and bark (*cortex Lycii radicis*) are the herbal raw material (Jin et al. 2013), although the therapeutic compounds are also contained in the seeds and leaves (Szafer et al. 1953; Wyk van and Wink 2008). *L. barbarum* fruits are characterised by a number of therapeutic properties, e.g. antiaging, protective, immunostimulant, energising, adaptogenic, anticancer, and antioxidant activity (Potterat 2010; Amagase and Farnsworth 2011) and are rich in many biologically active compounds such as specific polysaccharides, carotenoids, flavonoids, terpenoids, vitamins B and C, and the element germanium (Altintas et al. 2006; Li and Zhou 2007; Lin et al. 2009; Wang et al. 2010). Additionally, there are various triterpenes and steroids in the seeds, polyamines and peptides in the bark, and steroids in the leaves (Kremer 2011; Górnicka 2015). The most valuable components of wolfberry fruits are proteoglycans (glycoconjugates) forming a polysaccharide complex referred to as LBP (*Lycium barbarum* polysaccharides) and characterised by strong antioxidant, antiaging, neuroprotective, cytoprotective, anti-atherosclerotic, anti-fatigue, antitumour, and antidiabetic properties. They also contribute to biological endurance, increased metabolism, glucose control in diabetics, glaucoma control, and immunomodulation (Yi et al. 2013; Qiu et al. 2014; Zhang et al. 2014).

Although there are many reports of the content and chemical composition of phytochemicals contained in *Lycium barbarum* fruits, there is little scientific information about the fruit micromorphology, anatomy, and ultrastructure, as well as the location of bioactive compounds in the fruit cells and tissues. A study conducted by Bernardello (1986a) and Miller (2002) is the only report showing the layers in the pericarp structure and the number of seeds in *L. barbarum* drupes. In turn, Aguilar and Bernardello (2001) provided information on the size and weight of the fruit as well as the size and number of seeds in *L. cestroides.* Bernardello (1983, 1986b) described some aspects of the anatomical structure of the fruits of other *Lycium* species, i.e. *L. americanum*, *L. ameghinoi*, and *L. californicum*, in particular the structure of the hypodermis and the presence of the endocarp as well as the number of layers in the drupe pericarp.

Given the growing consumption and the wide spectrum of their health-enhancing properties, the aim of the study was to analyse the micromorphology, anatomy, and ultrastructure of *L. barbarum* fruits using histochemical and fluorescence techniques and to demonstrate which parts of the pericarp and/or seed accumulate several groups of biologically active compounds, in particular the health-promoting components. Knowledge of the distribution of secondary compounds can be helpful in enhancement of the therapeutic effect of these fruits and in preliminary assessment of their medicinal potential in the search for new pharmaceuticals. In addition, detailed anatomical studies are crucial for exploration of the determinants of fruit quality and can be useful for identification of diagnostic taxonomic traits (Ronse De Craene et al. 2000; Pak et al. 2001; Liu et al. 2010).

## Material and methods

Fully coloured and well-developed *Lycium barbarum* L. fruits were collected in the Botanical Garden of Maria Curie-Sklodowska University in Lublin, Poland (51° 15.629′ N 22° 30.975′ E) in the first decade of September 2016 and 2017. The species was identified using the classical Flora of China determination key for morphological validation, and the individuals were carefully investigated in terms of their floral and vegetative traits (Zheng-Yi and Raven 1994).

The fruits were examined with the use of light stereoscopic (SM), bright-field (LM), and fluorescence (FM) microscopes and under scanning (SEM) and transmission (TEM) electron microscopes.

## Stereoscopic and light bright microscopy

Preliminary observations and measurements of the length and width of fresh *L. barbarum* fruits and seeds were carried out using a SM equipped with a Nikon Coolpix 4500 camera.

For the LM analyses, 3 × 3 × 3 mm fragments of fruits with the skin (*n* = 5) were fixed, embedded in acrylic resin using the standard method applied for transmission electron microscopy (see below), and cut into 0.7-μm-thick semi-thin sections with glass knives in the Reichert Ultracut S microtome. For general histology examinations, the sections were stained with a 1% aqueous methylene blue-azure II solution (O’Brien and McCully 1981). Each fruit was analysed for the size of the epidermis and hypodermis cells, the thickness of the epidermis and hypodermis layers, the thickness of the cuticle layer and hypodermis walls, and the number of layers in the pericarp-forming parenchyma. The measurements were performed with the use of a light microscope Nikon 115 equipped with a calibrated ocular micrometre.

## Histochemistry and fluorescence

Hand-cut sections from fresh fruits and seeds were sampled using razor blades and viewed in water. Histochemical assays were applied to determine the content of primary and secondary metabolites in fruit and seed tissue (Table 1 and references wherein). Standard control procedures suggested by the different authors were applied simultaneously. All sections were observed under a Nikon Eclipse E200 light microscope (Nikon, Japan).

**Table 1:** Primary and secondary metabolites identified in the pericarp and seeds of *Lycium barbarum* by histochemical and fluorescence tests

Staining	Target compounds	Reference	Egzocarp	Mezocarp	Endocarp	Seed
Sudan III	Total lipids	Johansen 1940; Lison 1960	+	+	+	+
Sudan Red B	Total lipids	Brundrett et al. 1991	+	+	+	+
Sudan Black B	Total lipids	Pearse 1985	+	+	+	+
Nile Blue	Acidic lipids (oleoresins) Neutral lipids (essential oils)	Jensen 1962	+ −	+ −	+ −	+ +
Nadi reagent	Terpenoids (essential oils)	David and Carde 1964	+	−	+	−
Concentrated sulphuric acid	Sesquiterpenes	Geissmann and Griffin 1971; Cappelletti et al. 1986	+	−	+	−
Ruthenium Red	Acidic polysaccharides (mucilage, pectins)	Johansen 1940; Jensen 1962	+	+	+	+
Periodic acid - Schiff’s reagent (PAS)	Neutral polysaccharides	O’Brien and McCully 1981	+	+	+	+
Iodine iodide solution (IKI)	Starch Proteins	Johansen 1940	− +	− −	− +	+ +
Ferric chloride	Polyphenols	Johansen 1940	+		–	–
Potassium dichromate	Tannins	Gabe 1968	+		–	+
Phloroglucinol-HCl	Lignin	Johansen 1940	–	–	+	–
Wagner reagent	Alkaloids	Furr and Mahlberg 1981		+		+
Dragendorff reagent	Alkaloids	Svendsen and Verpoorte 1983		+		+
Neutral Red under UV	Lipids and essentials oils	Conn 1977; Lulai and Morgan 1992	−	+	+	+
Aluminium chloride under UV	Flavonoids	Charrière-Ladreix 1976	+	+	+	+
Magnesium acetate under UV	Flavonoids	Charrière-Ladreix 1976	+	+	+	+
Antimony trichloride under UV	Terpens contain steroids	Hardman and Sofowora 1972; Mace et al. 1974	+	−	+	+
UV (autofluorescence)	Polyphenols	Mabry et al. 1970	+	−	−	−

−, negative; +, positive

The fresh samples of fruits were also observed in FM to detect autofluorescence of polyphenols and the presence of lipids, flavonoids, and steroids with the use of different fluorochromes (Table 1 and references wherein) and filter sets; these included a Cy5 filter set (excitation light 590–650 nm and a barrier filter—wavelength 663–738 nm), a TRITC filter set (excitation light 525–565 nm and a barrier filter—wavelength 555–600 nm), a FITC filter set (excitation light 465–495 nm and a barrier filter—wavelength 515–555 nm), and a DAPI (excitation light 340–380, a barrier filter—wavelength 435–485). The observations were carried out under a Nikon 90i fluorescence microscope equipped with a digital camera (Nikon Fi1) and NIS-Elements Br 2 software.

## Scanning electron microscopy

The 3 × 3 × 2 mm fresh fruit samples with skin (*n* = 5) were not dried prior to the scanning electron microscopy (SEM) analyses, as the conventional fixation of material submitted to such observations can alter or remove lipids from the wax coating on the fruit surface (Konarska 2013a). After collection of the fruits from the bushes, the samples were cut out from the equatorial area perpendicular to the main axis of the fruit flesh and mounted carefully onto aluminium stubs with a double-sided carbon tape. The samples (3 × 3 × 2 mm) were coated with a 15-nm-thick layer of gold and examined under a TESCAN/VEGA LMU scanning electron microscope at an accelerating voltage of 10 kV.

## Transmission electron microscopy

Small sections (2 × 2 × 2 mm) of *L. barbarum* fruits (*n* = 5) were fixed in a mixture of 3.5% glutaraldehyde in 0.1 M phosphate buffer, pH 7.2, for 12 h at a temperature of 4 °C. Next, the sections for LM were washed three times in phosphate buffer and dehydrated in an ethanol series. For TEM, the permanent samples were additionally fixed in 1% OsO_4_ for 1.5 h and washed three times in distilled water. Next, the samples were rinsed in distilled water and dehydrated in a graded ethanol series. Then, the samples were embedded in LR white resin (LR White acrylic resin, medium grade, Sigma-Aldrich) and polymerised at 60 °C. Ultra-thin (70–80 nm) sections were cut with glass knives using a Reichert Ultracut S ultramicrotome and stained in a 0.5% aqueous solution of uranyl acetate in 0.5% acetic acid and lead citrate (Reynolds 1963). The ultrastructure was analysed under a Tesla BS 500 transmission electron microscope at an accelerating voltage of 120 kV.

## Results

**Fig. 1:**
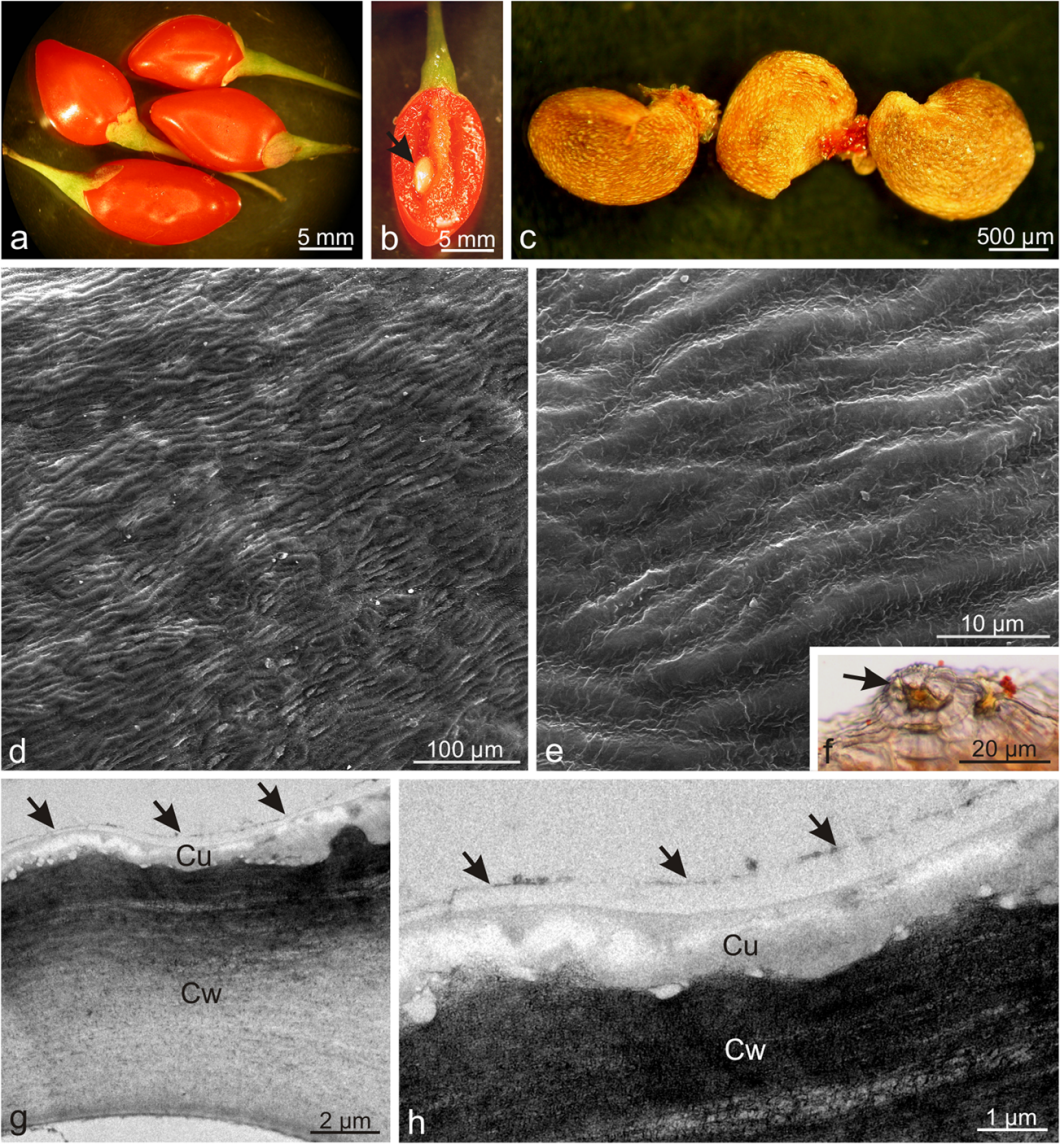
Morphology of *Lycium barbarum* drupes and drupelets as well as the ultrastructure of drupe epidermis cells. **a** Orange and elongated drupes. **b** Cross-section of wolfberry fruit. Visible two-seed chambers and a seed (*arrow*) in one of the chambers. **c** Flattened drupelets with a reticular surface. **d**, **e** Epidermis surface with numerous cuticular striae. **f** Stoma (*arrow*) located above the epidermis level. **g**, **h** Fragments of the epidermis cell wall with an amorphous cuticle and a layer of epicuticular waxes (*arrows*); *Cw* cell wall, *Cu* cuticle

**Fig. 2:**
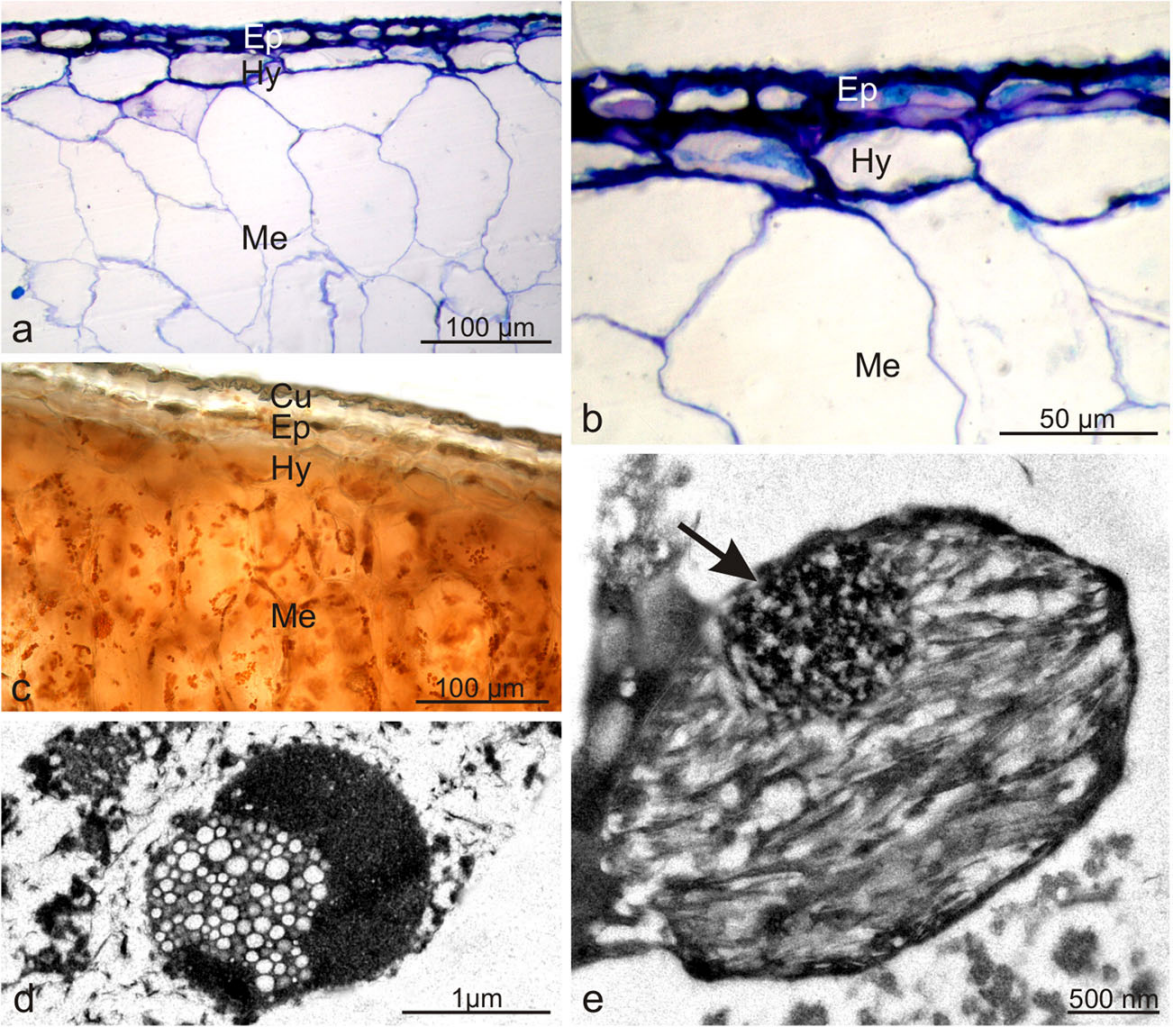
Anatomy and ultrastructure of the *L. barbarum* fruit pericarp. **a**–**c** Fragments of the fruit pericarp. Numerous chromoplasts visible in hypodermis and mesocarp cells (**c**). **d**, **e** Different types of chromoplasts in the hypodermis cells; vesicular chromoplast (**d**) and reticulotubular/fibrillar chromoplast with phytoferritine deposit (*arrow*) (**e**); *Cu* cuticle, *Ep* epidermis, *Hy* hypodermis, *Me* mesocarp

The elongated *Lycium barbarum* drupes were characterised by a varied length (0.7–1.5 cm) and width (3.5–7.5 mm) (Fig. 1a, b). The pericarp wall was composed of an exocarp (skin), mesocarp (flesh), and lignified endocarp surrounding two seeds. The yellowish seeds with a diameter of approx. 2.5–4 mm were oval and strongly flattened (Fig. 1c). During maturation, the fruits quickly became soft and susceptible to bruising. The 41–71.6-μm-thick fruit skin composed of one epidermis and one hypodermis layer was covered by a thin, delicately striated cuticle with a thickness of approx. 400–600 nm and amorphous structure (Table 2, Fig. 1 d, e, g, h). The epidermis exhibited sporadic stomata located slightly above the epidermis layer cells (Fig. 1f). TEM revealed a continuous 1/5-μm-thick film of amorphous epicuticular waxes on the surface of the cuticle (Fig. 1g, h). The epidermis cells exhibited large vacuoles and a thin layer of parietal cytoplasm with very small plastids containing numerous merging, irregularly shaped, and different-sized vesicles (not shown). The cross-sections of the epidermis cells visualised by LM were rectangular in the outline and exhibited a varied width (from 25.6 to 51.6 μm) and an almost uniform height (from 20.5 to 25.6 μm) (Fig. 2a, b). The outer and inner walls were thickened and had an average thickness of 5.6 and 4.5 μm, respectively. The hypodermis cells were characterised by a slightly thickened parietal cell wall adjacent to the epidermis and a periclinal wall adjacent to the mesocarp parenchyma (Fig. 2a, b) with an average thickness of 450 and 280 nm, respectively (Table 2). The size of the hypodermis cells varied; their width ranged from 28.1 to 84.4 μm and the height was in the range from 20.5 to 46 μm. The hypodermis cells visualised by LM and TEM contained large vacuoles, and cell nuclei and oval chromoplasts in various stages of development were visible in the cytoplasm. The initial stage of transformation of chloroplasts into chromoplasts was visible in some plastids. Such plastids were characterised by a lens shape and a largely preserved thylakoid system (not shown). Another type of chromoplasts, i.e. the so-called vesicular chromoplasts, exhibited many different-sized vesicles usually with electron-transparent content (Fig. 2d). The largest group of plastids comprised oval-shaped chromoplasts containing reticulotubular/fibrillar carotenoid pigments (Fig. 2e). There were no thylakoids in this type of plastids, but phytoferritine deposits were frequently visible as granular osmiophilic bodies (Fig. 2e). Degraded protoplast components, usually remnants of plasmatic membranes, were frequently observed in the hypodermis cells. The multi-layer mesocarp located under the skin was composed of relatively large, oval or radially elongated parenchyma cells with thin walls (Fig. 2a–c). Intercellular spaces were visible between the cells. The mesocarp cells contained many chromoplasts similar to those observed in the hypodermis (Fig. 2c).

**Table 2:** Comparison of the anatomical characteristics of the skin of *Lycium barbarum* and other fruit species

Species	Thickness of cuticle layer (μm)	Thickness of hypodermis layer (μm)	Thickness of hypodermis cell wall (μm)	Thickness of skin (μm) (epidermis + hypodermis)	Reference
*Lycium barbarum*	0.6	35.0	0.28–0.45	59.6	This study
*Vaccinium corymbosum*	3.3	38.7–56.7	4.4–5.2	54.6–77.5	Konarska 2015a, b
*Pyrus communis*	8.9–11.5	46.8–61.3	7.5–26.0	57.7–74.7	Konarska 2013b
*Viburnum opulus*	5.3	80.0	17.8	109.6	Konarska and Domaciuk 2018
*Viburnum lantana*	6.5	72.7	6.7	113.5	
*Prunus domestica*	4.6–4.9	76.0–110.0	7.6–8.4	109.0–129.0	Konarska 2015c
*Malus domestica*	12.3–17.2	75.4–95.6	14–17	116.8–125.4	Konarska 2013a, 2014

**Fig. 3:**
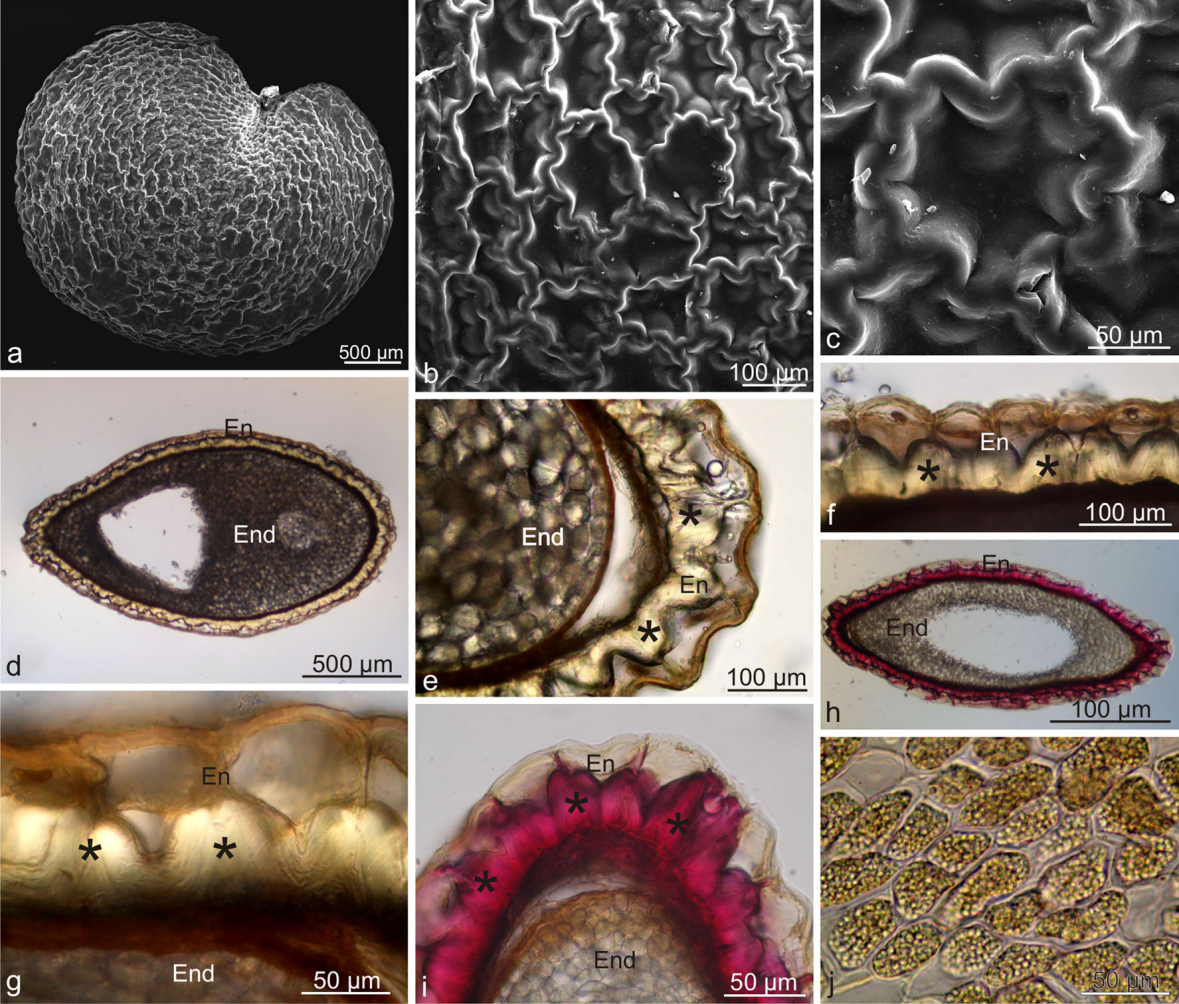
Micromorphology and anatomy of a *L. barbarum* drupelet. **a**–**c** Drupelet surface with reticulate-puzzle architecture. **d** Cross-section of an endocarp and seed. **e**–**g** Fragments of cross-sections of an endocarp and seed. Notice the thickened and lignified cell walls of the endocarp (*asterisks*). **h**, **i** Cross-section of a drupelet stained with phloroglucinol with HCl. **j** Endosperm cells; *En* endocarp, *End* endosperm

The single-layered endocarp surrounding endosperm seeds was composed of stone cells with undulated, unevenly thickened, lignified secondary walls forming specific ‘cavernulous’ reticulate-puzzle architecture on the seed surface (Fig. 3a–c). LM visualised lignin thickenings with intensive pink stain as a result of the reaction with phloroglucinol with hydrochloric acid in the periclinal walls adjacent to the seed testa and partly in anticlinal walls, where they were usually triangular or trapezoidal (Fig. 3d–j). Their structure exhibited successive layers formed by adcrustation. The periclinal endocarp walls adjacent to the mesocarp were much thinner and devoid of lignin (Fig. 3e–h). Despite the considerable lignification of the walls, the endocarp cells had living protoplasts. In turn, cells forming the seed endosperm were oval and had thickened cellulose-pectin walls (Fig. 3j). Calcium oxalate crystals with the shape of truncated pyramids were observed in the endosperm cells (not shown).

**Fig. 4:**
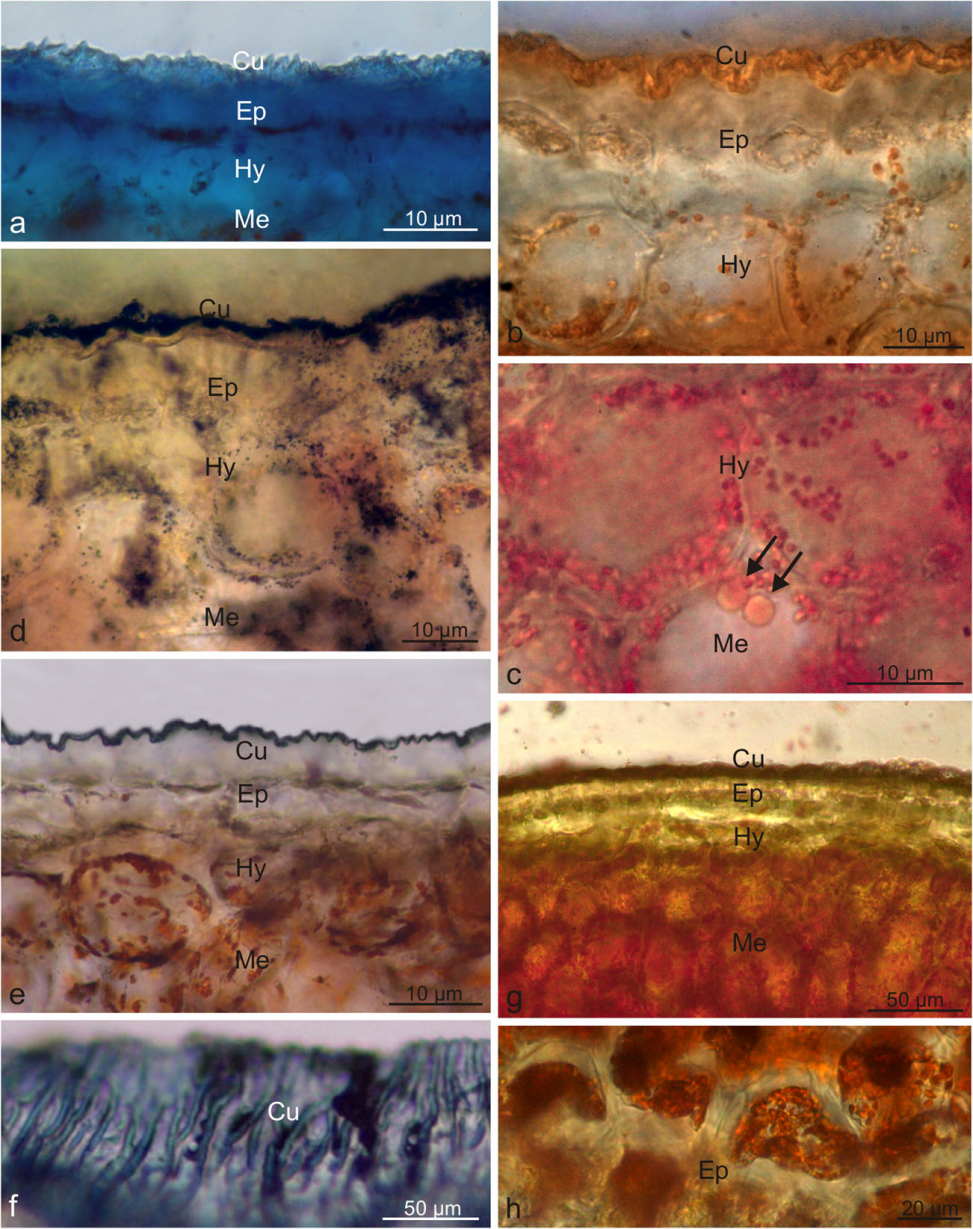
Fresh cross-sections across the *L. barbarum* pericarp subjected to histochemical tests. **a** Staining with Nile Blue. **b** Staining with Sudan III. **c** Staining with Sudan Red B (top view). **d** Staining with Sudan Black B. **e**, **f** Staining with Nadi reagent. **f** Top view. **g**, **h** Staining with conc. sulphuric acid. **h** Top view; *Cu* cuticle, *Ep* epidermis, *Hy* hypodermis, *Me* mesocarp, *arrows*: lipid droplets

**Fig. 5:**
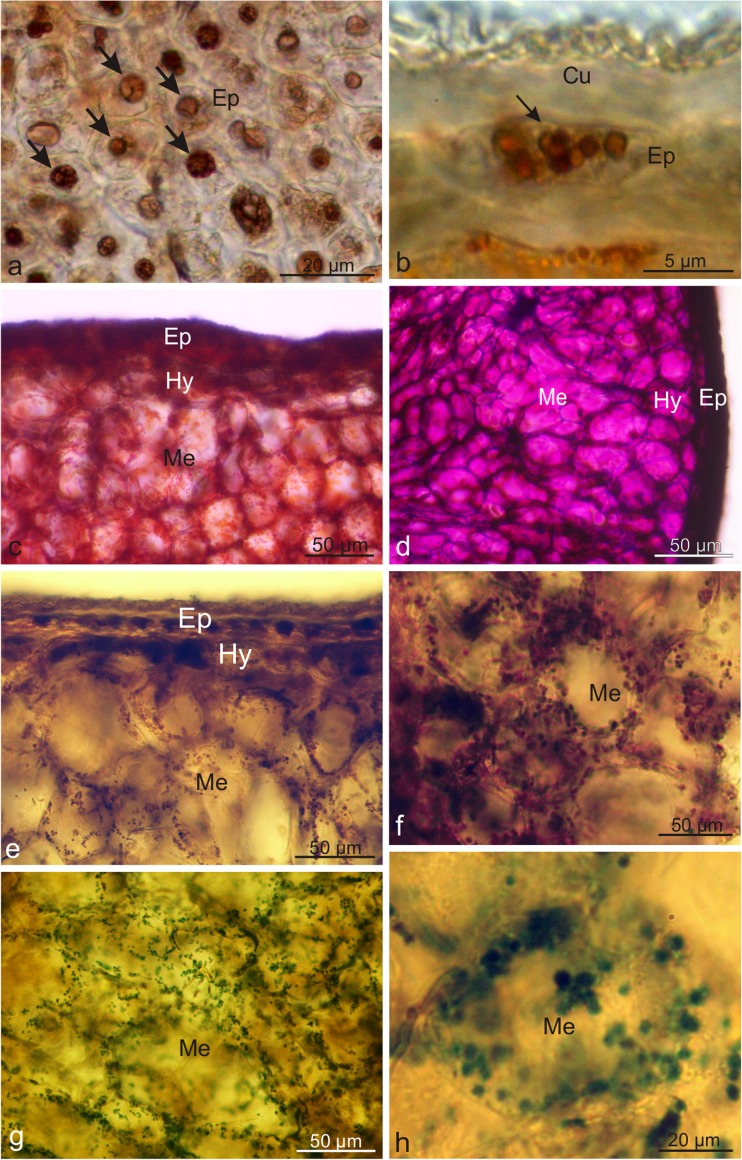
Fresh cross-sections across the *L. barbarum* pericarp subjected to histochemical tests. **a** Staining with potassium dichromate (top view). **b** Staining with ferric chloride. **c** Staining with Ruthenium Red. **d** Staining with Schiff’s reagent. **e** Staining with Wagner reagent. **f** Staining with Dragendorff reagent. **g**, **h** Staining with IKI solution; *Cu* cuticle, *Ep* epidermis, *Hy* hypodermis, *Me* mesocarp, *arrow*: phenolic deposits

The results of the histochemical assays showed the presence of lipid, terpenoids, polysaccharides, and polyphenols in the cuticle and epicuticular waxes covering the wolfberry fruits (Table 2). Lipids contained in the epidermis and hypodermis cell walls were stained blue in the presence of Nile Blue, whereas lipids present in the cuticle were turquoise (Fig. 4a). After the reaction with Sudan III, Sudan Red B, and Sudan Black B, cuticle, large lipid droplets and/or small globoids present in the epidermis, hypodermis, and parenchyma cells were stained orange, orange-red, and black, respectively (Fig. 4b–d). The blue colour of the cuticle after application of the Nadi reagent indicated the presence of terpenoids (Fig. 4e, f). In the presence of concentrated sulphuric acid, sesquiterpenes located in the pericarp, and mainly in the epidermis and hypodermis cell walls, were stained yellow (Fig. 4g, h). The epidermis and hypodermis cells of the drupes exhibited spherical deposits of tannins and phenolic compounds with various sizes and structures; they were stained brown when treated with potassium dichromate and ferric chloride (Fig. 5a, b). The use of Ruthenium Red confirmed the presence of polysaccharides in the skin cells and in the cell walls of the pericarp layers (Fig. 5c). In turn, in the reaction with Schiff’s reagent, polysaccharides present in the cell protoplasts in all parts of the pericarp exhibited an intense cyclamen colour while cell wall polysaccharides were purple (Fig. 5d). The Wagner and Dragendorff reagents produced dark brown stain of alkaloids contained in the epidermis and in the cells of the parenchyma, especially in its deeper layers (Fig. 5e, f). After application of the Lugol’s iodine solution, the chromoplasts located in the pericarp cells were stained dark turquoise (Fig. 5g, h).

**Fig. 6:**
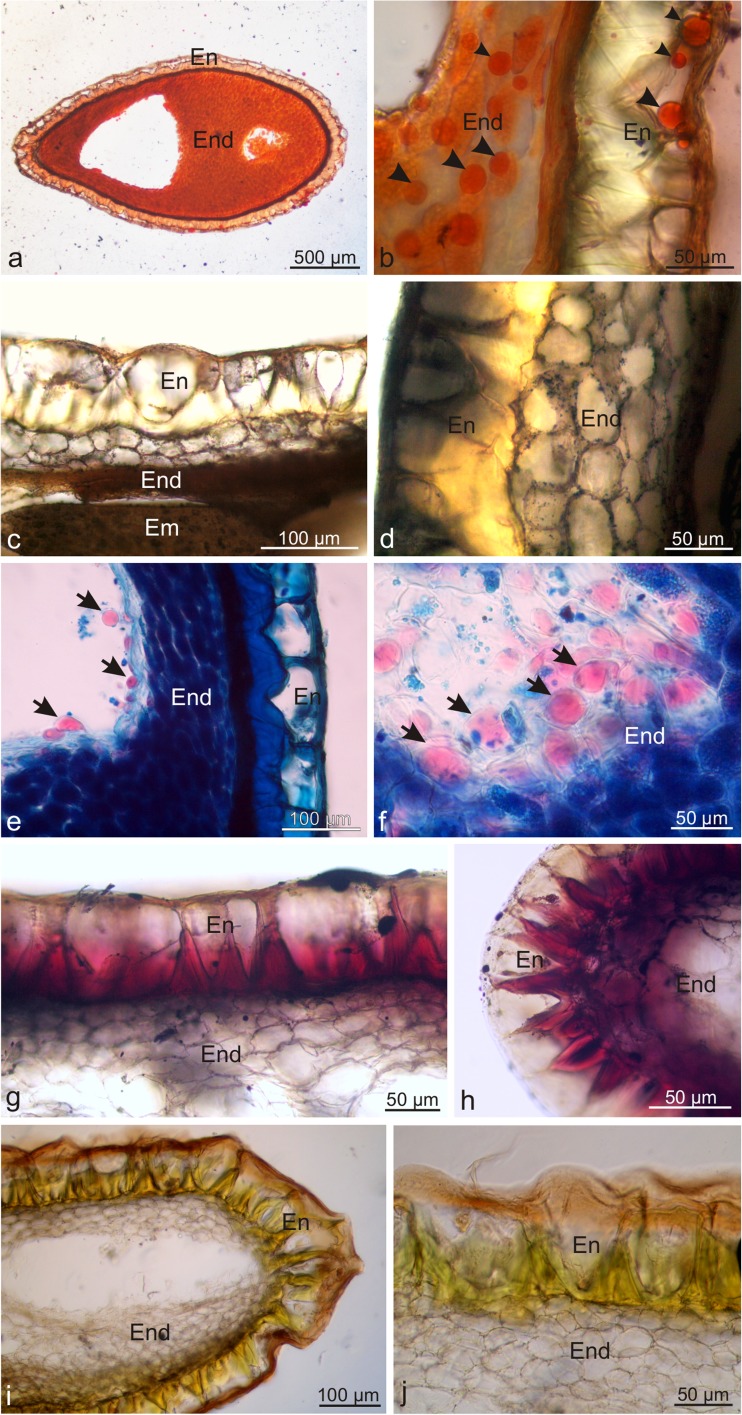
Fresh cross-sections across *L. barbarum* drupelets subjected to histochemical tests. **a**, **b** Staining with Sudan Red B. **c**, **d** Staining with Sudan Black B. **e**, **f** Staining with Nile Blue. **g**, **h** Staining with Nadi reagent. **i**, **j** Staining with conc. sulphuric acid; *En* endocarp, *End* endosperm, *Em* embryo, *arrows*: droplets of essential oils, *arrowheads*: oil droplets

**Fig. 7:**
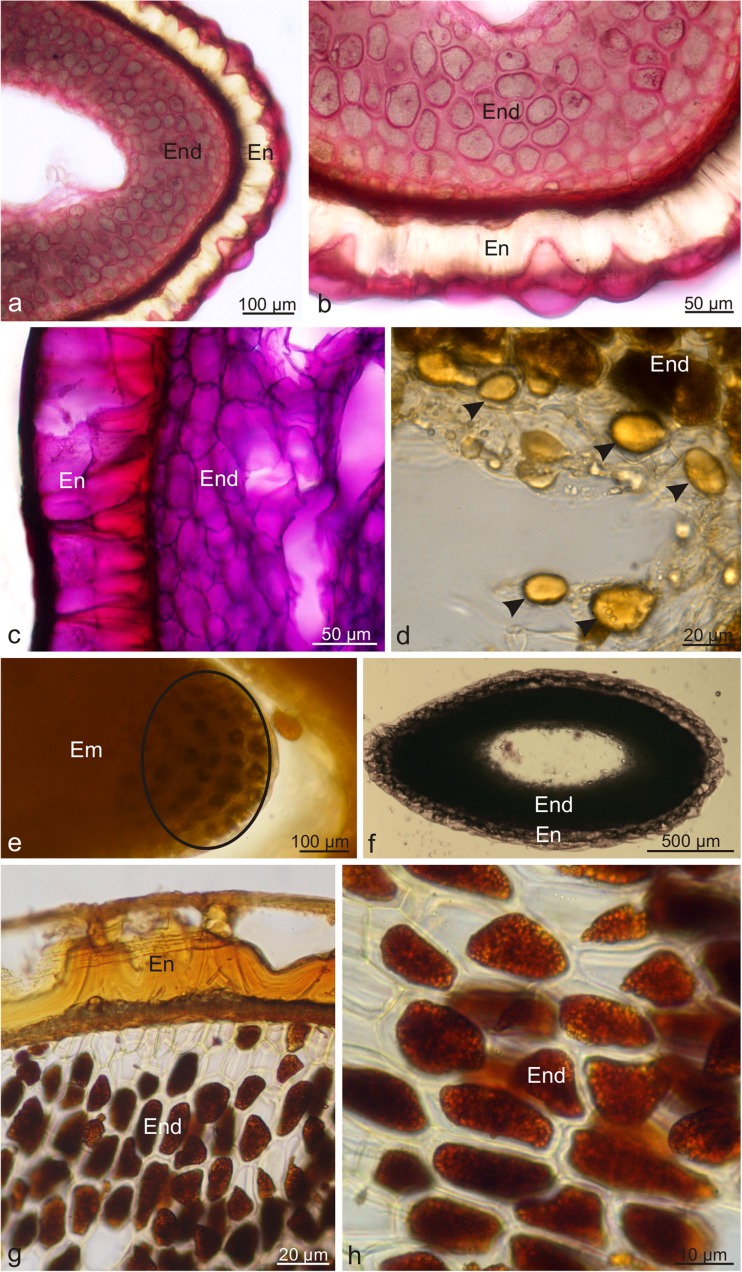
Fresh cross-sections across *L. barbarum* drupelets subjected to histochemical tests. **a**–**b** Staining with Ruthenium Red. **c** Staining with Schiff’s reagent. **d**, **e** Staining with IKI solution. **f** Staining with potassium dichromate. **g** Staining with Wagner reagent. **h** Staining with Dragendorff reagent; *En* endocarp, *End* endosperm, *Em* embryo, *arrowheads*: aleurone grains, *circle*: cells with starch grains

The endocarp and seeds of the wolfberry drupes contained lipids, terpenoids, polysaccharides, polyphenols, flavonoids, starch, proteins, and alkaloids (Table 1). The numerous lipid droplets visible in the endocarp and endosperm cells were orange-red after the treatment with Sudan III and Red B, whereas the application of Sudan Black B revealed tiny, black lipid globoids in the endocarp, endosperm, and embryo cells (Fig. 6a–d). Nile blue stained essential oils present in the endosperm cells intensely pink, whereas acidic lipids in the walls and protoplasts of the endocarp and endosperm cells had a navy blue colour (Fig. 6e, f). In turn, essential oils contained in the lignified endocarp walls were cyclamen pink after staining with the Nadi reagent (Fig. 6g, h). Sesquiterpenes accumulated in the endocarp cell walls were stained yellow in the presence of concentrated sulphuric acid (Fig. 6i, j). Ruthenium Red showed the presence of pink and red stained polysaccharides in the walls and protoplasts of the endocarp cells, seed testa, and endosperm cell walls (Fig. 7a, b). Similarly, the PAS reaction confirmed the presence of polysaccharides in the protoplasts and lignified endocarp cell walls as well as endosperm cells (Fig. 7c). Additionally, the endosperm cells exhibited large, oval aleurone grains filled with amorphous protein, which stained yellow in the presence of the IKI solution (Fig. 7d). The treatment with Lugol’s liquid resulted in dark blue staining of starch grains present in the embryo cells (Fig. 7e). Brown-stained tannins were localised in the endocarp and endosperm cells in the reaction with potassium dichromate (Fig. 7i). The Wagner and Dragendorff reagents showed the presence of alkaloids in the brown-stained protoplasts in the endosperm cells (Fig. 7g, h).

**Fig. 8:**
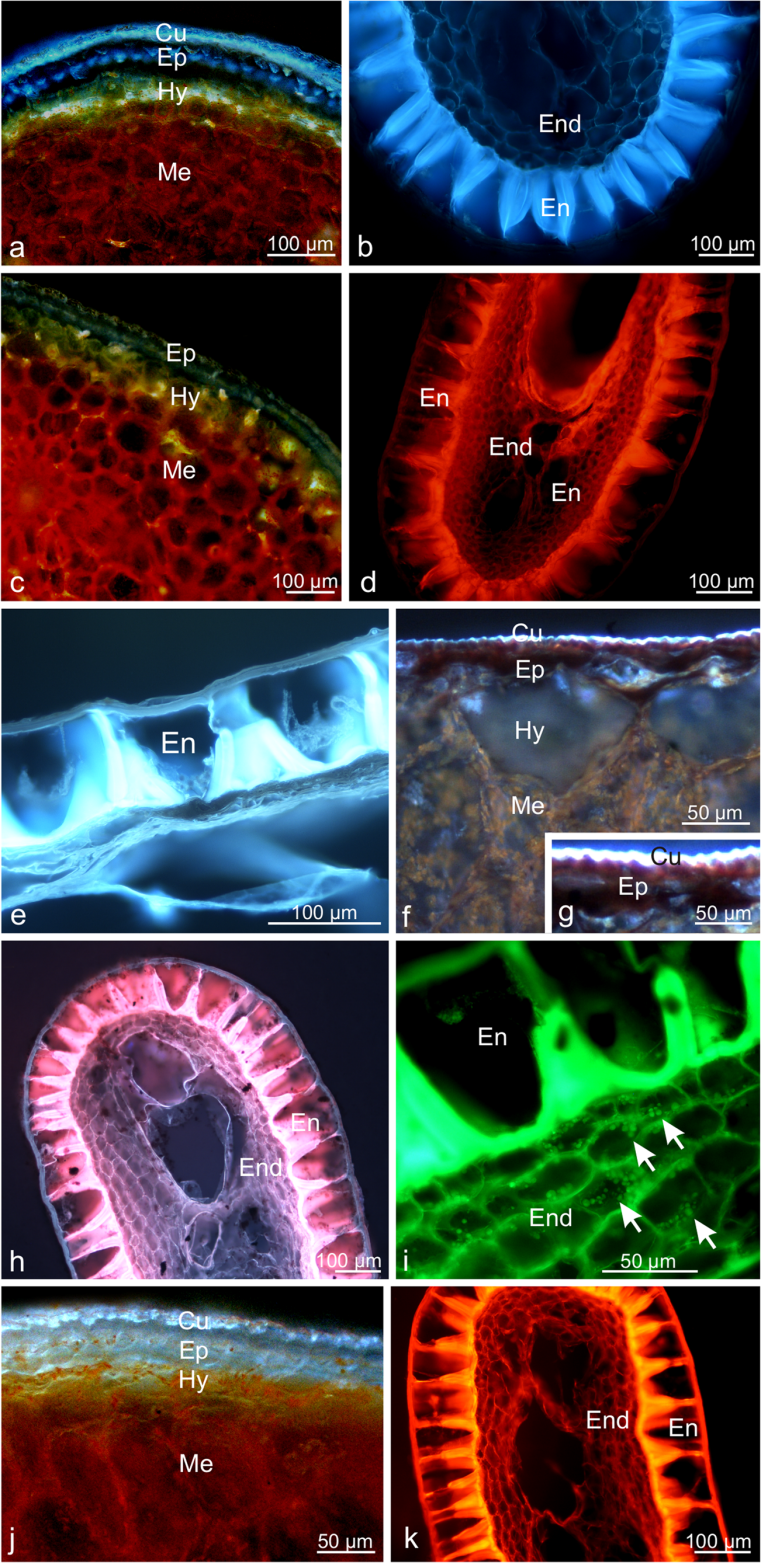
Fresh cross-sections across the *L. barbarum* pericarp and drupelets under a fluorescence microscope. **a**, **b** Fluorescence of steroids in the drupe skin (**a**) and in the endocarp and endosperm cell walls (**b**) in the Cy5 filter set after application of antimony trichloride. **c**, **d** Fluorescence of flavonoids in the skin and mesocarp cells in the Cy5 filter set (**c**) and in the endocarp and edosperm cells in the TRITC filter set (**d**) after application of magnesium acetate. **e** Fluorescence of flavonoids in the endocarp and edosperm cells in the Cy5 filter set after application of aluminium chloride. **f**–**i** Fluorescence of essentials oils in the cuticle (**f**, **g**) and in endocarp cell walls (**h**) in the Cy5 filter set as well as fluorescence of lipid droplets in the endosperm cells in the FITC filter set (**i)** after application of Neutral Red. **j**, **k A**utofluorescence the phenolic compounds in skin cells in the Cy5 filter set (**j**) and in the lignified endocarp cell walls in the TRITC filter set (**k**); *arrows:* lipid droplets

The fluorescence microscopy observations under the treatment with the antimony trichloride fluorochrome in the Cy5 filter set confirmed the presence of steroid-containing terpenes (light blue fluorescence) in the drupe skin (especially in the cuticle and cell walls) and in the endocarp and endosperm cell walls (Table 1, Fig. 8a, b). Moreover, in the presence of the aluminium chloride and magnesium acetate fluorochromes, there was visible light yellow (in the Cy5 and DAPI filter sets) and light red (in the TRITC filter set) fluorescence of flavonoids accumulated in the skin and mesocarp cells and in the endocarp and endosperm cells of the wolfberry fruits (Fig. 8c–e). In turn, neutral red induced light blue (in the Cy5 filter set) or light green (in the FITC filter set) secondary fluorescence of essentials oils contained in the cuticle and endocarp cell walls as well as lipid droplets in the endosperm cells (Fig. 8f–i). Additionally, strong light blue (in the Cy5 filter set) autofluorescence of the skin cells and light orange (in the TRITC filter set) autofluorescence of the lignified endocarp cell wall was observed, indicating the presence of some phenolic compounds (Fig. 8j, k).

## Discussion

Based on the microscopic observations (reaction of phloroglucinol with hydrochloric acid), the author of this paper has shown that the *Lycium barbarum* fruit is a two-seeded drupe with lignified cell walls of the seed-surrounding endocarp. Researchers have contrasting opinions on whether the fruit of the analysed species is a drupe or a berry. Bernardello (1983, 1986a) and Olmstead et al. (2008) also argue that the fruit of many *Lycium* species are drupes with a sclerenchymatic endocarp, whereas Miller (2002) claims that the representatives of the genus *Lycium* have a berry-like fruit differing in the degree of endocarp induration and the number of seeds. The analysed *L. barbarum* fruits have 2 seeds, whereas Bernardello (1986a) and Aguilar and Bernardello (2001) have shown that fruits of other *Lycium* species (*L. ciliatum*, *L. chilense*, and *L. cestroides*) are multi-ovuled and multi-seeded. As reported by various researchers, the number of seeds in the genus *Lycium* can be varied: it is usually 2 or 4 and, less frequently, 8, 10 or more (Miller 2002; Levin and Miller 2005). The number of seeds may be an important taxonomic trait, although the seed number can be determined by not only the genetic factor but also unfavourable weather conditions prevailing during the period of flowering and flower visiting by pollinating entomofauna. The author of this work have observed that the analysed *Lycium barbarum* shrubs produced an evidently lower number of fruits in the dry and hot summer of 2017 than in the less hot and dry summer of 2016.

During the investigations, it was noted that ripe wolfberry fruits quickly lost their attractiveness: their surface was wrinkled and damaged by indentation and bruises. Such symptoms may be associated with the thin and delicate skin covering the drupe. Its thickness was approximately twofold lower than that of apple, plum, or viburnum skin and similar to the skin of blueberries and pears (Table 2 and reference wherein). The fruits of blueberry and early pear varieties represent short shelf life fruits sensitive to mechanical damage. Wyk van and Wink (2008) and Górnicka (2015) have confirmed that ripe *L. barbarum* fruits are prone to mechanical damage and are non-durable; therefore, they are not picked by hand but gently shaken off. The surface of the *L. barbarum* fruits was covered by a thin striated cuticle with amorphous structure. As shown by Knoche et al. (2000), the thickness of the cuticle is not correlated with cuticular water permeability, in contrast to its ultrastructure, which can accelerate or limit transpiration and fruit wilting. Various researchers have reported that an amorphous cuticle promotes water evaporation from the fruit interior and contributes to rapid wilting. In turn, a cuticle with a lamellar structure, which is characteristic for e.g. apple fruits, limits transpiration to the greatest extent (Peschel et al. 2003; Jeffree 2006). Chiarini and Barboza (2007) and Pabón-Mora and Litt (2011) have shown that the cuticle thickness in the berries or drupes of other representatives of the family Solanaceae may vary from a relatively thick cuticle in *Lycopersicon* to that in *Iochroma*, which is immeasurable by light microscopy.

A great role in fruit life is assigned to the thickness of the wax layer and the type of epicuticular waxes present on the fruit surface. A relatively thin layer of amorphous waxes was observed in the *L. barbarum* fruits, but no form of crystalline wax was detected, which undoubtedly accelerates transpiration of the fruits and shortens their shelf life. Many researchers have also reported that wax crystallites present on the fruit surface extensively limit water evaporation and protect fruits against adverse biotic and abiotic factors (Veraverbeke et al. 2001; Solovchenko and Merzyak 2003; Knoche 2015). In contrast, the amorphous wax form, similar to the amorphous cuticle, is the most permeable type of wax contributing to rapid water loss. This type of epicuticular waxes has been observed on the fruit surface of such taxa as *Capsicum annuum*, *Prunus avium*, or *Viburnum opulus* (Lownds et al. 1993; Hunsche and Noga 2011; Konarska and Domaciuk 2018).

The hypodermis in the *L. barbarum* drupes is composed of one layer of collenchyma cells with slightly thickened periclinal walls. The inconsiderable thickness of the hypodermis wall and its relatively thin layer are the other features that make the fruits delicate and soft. The author of the present study has demonstrated that the hypodermis walls in the *Lycium barbarum* drupes are several times thinner than in the *Vaccinium corymbosum* berries, which are similarly considered delicate and short-lived, and several tenfold thinner than in the fruits of *Malus domestica* or *Viburnum opulus*, which are characterised by substantial shelf life (Table 2 and reference wherein). In investigations of fruits of several other *Lycium* species, Bernardello (1983) did not distinguish a hypodermis layer in any of the species. The author did not measure and compare the thickness of the cell walls in the pericarp tissues in the examined *Lycium* drupes. However, the presence of a collenchyma layer under the fruit epidermis has been reported in many other Solanaceae species, e.g. in *Capsicum* and *Athenaea* (Filippa and Bernardello 1992), *Iochroma* (Pabón-Mora and Litt 2011), and many *Solanum* species (Dottori and Cosa 1999, 2007; Charini and Barboza 2007, 2009). Various authors suggest that the textural characteristics of skin cells (e.g. the thickness of the skin and the presence of mechanical tissue as well as the thickness of the cell walls and the degree of cell to cell contact) determine fruit susceptibility to mechanical damage, which reduces consumer value and shortens their shelf life (Allan-Wojtas et al. 2001; Klima Johnson et al. 2011).

The author of the present paper has distinguished several layers of parenchyma forming the mesocarp and one layer of a lignified endocarp surrounding the seeds in the *Lycium* fruit. Bernardello (1983) reported that the pericarp wall in the fruits of other *Lycium* species (*L. americanum*, *L. ameghinoi*, *L. californicum*), similar to the ovary wall, was always composed of eight layers and the endocarp was made of sclerenchyma. Similar observations were reported by Chiang-Cabrera (1981), who showed the presence of an indurated endocarp in the fruits of four other *Lycium* species: *L. cooperi*, *L. macrodon*, *L. piiherulum*, and *L. schaffneri.* The *L. barbarum* endocarp exhibited a specific ‘cavernulous’ reticulate-puzzle sculpture, which was probably related to the heterogeneous chemical structure of the cell walls in this layer. The anticlinal walls of the endocarp were formed of lignin, which ensured their stiffness, whereas the elastic periclinal walls adjacent to the mesocarp contained cellulose and pectins. The author of the study observed that such structure of the endocarp cell walls ensures their stiffness, but does not lead to the death of protoplast, in which metabolites (lipids, polysaccharides) can accumulate. A similar ornamentation of the seed surface was described in several other Solanaceae species (*Tubocapsicum*, *Aureliana*, *Withania*, *Hyoscyamus*) by D’Arcy et al. (2001) and Kaya et al. (2016). The structure of endocarp cells and/or seed testa can be an important taxonomic characteristics, as proved by Gunn and Gaffney (1974) and Axelius (1992) in various representatives of the family Solanaceae.

The intense orange colour of *L. barbarum* fruits was associated with the presence of numerous carotenoid-containing chromoplasts in the pericarp cells. As reported by Miller (2002), the colour of fruits in different *Lycium* species can range from yellow through orange or red to green or brown. The presence of thylakoids in many plastids of the *L. barbarum* pericarp indicates transformation of chloroplasts present in green fruits into chromoplasts specific for mature fruits. In turn, fully developed *L. barbarum* chromoplasts exhibited numerous carotenoid-filled plastoglobules or the carotenoids had a reticulotubular/fibrillar form. The chromoplasts also contained spherical phytoferritine deposits, i.e. complexes of iron with protein. As shown by literature data, phytoferritine deposits are a non-toxic form of accumulation and storage of iron; they can subsequently be used in the synthesis of carotenoids and serve an important function during fruit development and in stress conditions (Simpson et al. 1975; Briat 1996; Di Fabio and Parraga 2016). Various types of chromoplasts, i.e. globular, membranous, tubular, fibrillar, or crystalline forms observed during chromoplastogenesis, have been described in the fruits of family Solanaceae representatives (e.g. Kilcrease et al. 2013; Suzuki et al. 2015). As shown by Rosso (1967), several different forms of chromoplasts can be present simultaneously in same species. In turn, as suggested by Ljubesić et al. (1991), there are many different pigment-containing structures even in the same chromoplast. Moreover, Schweiggert et al. (2011) and Jeffery et al. (2012) indicate that the type of the chromoplast structure depends on the type of carotenoid molecules accumulated in these organelles and determines the bioavailability of carotenoids during human digestion.

The histochemical assays and fluorescence microscopy observations have revealed that the *L. barbarum* fruits are a source of many various secondary metabolites. Besides carotenoids, polysaccharides (LBP), terpenoids (essential oils, steroids, sesquiterpenes, oleoresins), polyphenols (tannins and flavonoids), and alkaloids were accumulated in the pericarp and seed of the drupes.

The presence of polysaccharides other than starch and cellulose in the protoplasts of the mesocarp cells in the *Lycium barbarum* drupes was confirmed by the reactions with Ruthenium Red and Schiff’s reagent. Currently, there are no histochemical methods that would confirm the presence of specific polysaccharides in wolfberry fruits, i.e. the so-called LBP. Nevertheless, the author observed that the wolfberry pericarp chromoplasts exhibited an untypical turquoise-green colour when stained with Lugol’s iodine. Probably, intermediate polysaccharide fractions such as rhamnose, arabinose, mannose and/or LBP-forming proteoglycans were accumulated in the chromoplasts, bearing in mind that chromoplasts were shown by Neuhaus and Emes (2000) and Barsan et al. (2010) to be involved not only in the synthesis of lipids and aromatic compounds, but also in the production of sugars.

The terpenoids in the *L. barbarum* drupes were primarily accumulated in the fruit skin and endocarp. A special role is attributed to terpenoids present on the surface of the epidermis, i.e. in the cuticle and wax, and in lignified endocarp walls. Given their specific smell, these compounds can contribute to the dispersal of fruits and seeds by attracting consumers and/or they can repel frugivores. Additionally, essential oils exhibit strong antibacterial and antifungal activity and are toxic to nematodes and molluscs (Nishida 2002; Gershenzon and Dudareva 2007). The presence of 11 different terpenoids and over 50 sterols and steroids has been demonstrated in *L. barbarum* fruits (Qian et al. 2017).

Tannins and flavonoids are phenolic compounds that were accumulated in the exocarp, endocarp, and endosperm of the wolfberry drupes. Tannin deposits detected in the skin had a form of globules with varied structure and size, i.e. so-called proanthocyanidins. This type of tannin deposits present in the skin of various fruits has also been reported by other authors (Hammouda et al. 2014; Tessmer et al. 2014; Konarska and Domaciuk 2018). Phenolic compounds are valuable antioxidants with an ability to chelate metal ions and scavenge free radicals thus enhancing the plant defence mechanism (Sengul et al. 2009; Karamian and Ghasemlou 2013). Moreover, similar to terpenoids and flavonoids, tannins can deter herbivores and protect plants against pathogens (Lattanzio et al. 2006; He et al. 2015; Tessmer et al. 2016). They also determine the attractiveness to fruit and seed dispersers (Havsteen 2002; Cazetta et al. 2008) and protect the fruit against the harmful effects of UV-B radiation (Robson et al. 2015; Siipola et al. 2015).

The *L. barbarum* drupes contained alkaloids, as confirmed by the histochemical assays based on the Wagner and Dragendorff reagents. These compounds were present in the exocarp and endosperm of the seeds. As in the case of other repellent compounds (tannins, flavonoids), the location of alkaloids in sensitive fruit layers exposed to pathogens or herbivores may allow the plant to protect the propagules and to reproduce effectively. A similar observation was done by Adler (2000), who stated that alkaloids not only can increase plant fitness by reducing herbivore attack, but also indirectly increase the lifetime seed production. The toxicity of *L. barbarum* fruits has aroused controversy for many years. For a long time, the fruits were believed to be highly toxic due to the content of atropine, hyoscyamine, and scopolamine (Harsh 1989). However, the quantitative investigations conducted by Adams et al. (2006) and Wang (2006) have questioned the toxicity of *L. barbarum* fruit although the presence of these alkaloids has been confirmed. Recent research has evidenced that wolfberry fruits are a source of approximately 20 piperidine, pyrrole, spermine, and tropane alkaloids (Qian et al. 2017), but they are present at a very low, non-toxic level (Yong 2005).

## Conclusions

*L. barbarum* drupes are characterised by a short shelf life and susceptibility to mechanical damage. These first examinations of the wolfberry fruit microstructure demonstrated that the reduced quality and durability of drupes is determined by their thin skin, amorphous cuticle, thin layer of amorphous epicuticular waxes, and thin layer of the hypodermis with slightly thickened walls. Wolfberry fruits are regarded as functional food and a source of various types of bioactive compounds located in all pericarp layers and seeds, especially in the skin and endocarp of the drupes. Metabolites that are accumulated in *L. barbarum* drupes and attributed special phytotherapeutic importance include specific polysaccharides (LBP), carotenoids, terpenoids (essential oils, sesquiterpenes, and steroids), polyphenols (tannins and flavonoids), and alkaloids. The unique microstructural and histochemical analyses presented in this paper are a valuable addition to phytochemical investigations, as they facilitate detailed determination of the sites/regions of synthesis and/or storage of pharmacologically active compounds at the tissue level and help to understand the relationship between the tissue structure and function as well as the chemical nature of the tissues. The knowledge of the location of bioactive plant metabolites facilitates identification of therapeutically promising and valuable compounds applied in folk herbal and traditional medicine. Furthermore, the knowledge of the fruit microstructure, in particular the analyses of the differentiation of plastid structure and pigmentation, may contribute to a better understanding of the biogenesis of these fruits as well as their developmental and environmental regulation.
